# Activation of the Interferon Pathway is Dependent Upon Autoantibodies in African-American SLE Patients, but Not in European-American SLE Patients

**DOI:** 10.3389/fimmu.2013.00309

**Published:** 2013-10-01

**Authors:** Kichul Ko, Yelena Koldobskaya, Elizabeth Rosenzweig, Timothy B. Niewold

**Affiliations:** ^1^Section of Rheumatology, Gwen Knapp Center for Lupus and Immunology Research, University of Chicago, Chicago IL, USA; ^2^Department of Immunology, Division of Rheumatology, Mayo Clinic, Rochester, MN, USA

**Keywords:** systemic lupus erythematosus, interferon alpha, autoantibodies, ancestral background, interferon gamma

## Abstract

**Background:** In systemic lupus erythematosus (SLE), antibodies directed at RNA-binding proteins (anti-RBP) are associated with high serum type I interferon (IFN), which plays an important role in SLE pathogenesis. African-Americans (AA) are more likely to develop SLE, and SLE is also more severe in this population. We hypothesized that peripheral blood gene expression patterns would differ between AA and European-American (EA) SLE patients, and between those with anti-RBP antibodies and those who lack these antibodies.

**Methods:** Whole blood RNA from 33 female SLE patients and 16 matched female controls from AA and EA ancestral backgrounds was analyzed on Affymetrix Gene 1.0 ST gene expression arrays. Ingenuity Pathway Analysis was used to compare the top differentially expressed canonical pathways amongst the sample groups. An independent cohort of 116 SLE patients was used to replicate findings using quantitative real-time PCR (qPCR).

**Results:** Both AA and EA patients with positive anti-RBP antibodies showed over-expression of similar IFN-related canonical pathways, such as IFN Signaling (*P* = 1.3 × 10^−7^ and 6.3 × 10^−11^ in AA vs. EA respectively), Antigen Presenting Pathway (*P* = 1.8 × 10^−5^ and 2.5 × 10^−6^), and a number of pattern recognition receptor pathways. In anti-RBP negative (RBP−) patients, EA subjects demonstrated similar IFN-related pathway activation, whereas no IFN-related pathways were detected in RBP−AA patients. qPCR validation confirmed similar results.

**Conclusion:** Our data show that IFN-induced gene expression is completely dependent on the presence of autoantibodies in AA SLE patients but not in EA patients. This molecular heterogeneity suggests differences in IFN-pathway activation between ancestral backgrounds in SLE. This heterogeneity may be clinically important, as therapeutics targeting this pathway are being developed.

## Introduction

Systemic lupus erythematosus (SLE) is a heterogeneous disease characterized by complex genetic contributions and activation of a number of immune system pathways (1–3). Recent advances in human genetic studies have helped us better understand the immunopathogenesis of the disorder (4, 5). Multiple candidate gene association studies and genome wide association studies have led to discovery of more than 30 susceptibility loci throughout the whole genome, most of which are involved in three main pathways in lupus pathogenesis: abnormal clearance of nuclear debris and immune complexes, over-activation of innate immune system through Toll-like receptor (TLR) and type I interferon (IFN) signaling, and aberrant adaptive immune response through B and T cell signaling (6, 7). Moreover, gene expression microarray studies have been instrumental in defining important aspects of the complex immunological pathogenesis in human subjects (8, 9). Several gene expression analyses in SLE have found up-regulation of IFN-inducible genes (IFIGs) in more than 50% of patients (10–13), and others have shown differential expression of genes involved in several pathways including inflammation, apoptosis, DNA repair, and T cell activation (12, 14–17).

Interferon-α is a pleiotropic type I IFN which plays a key pathogenic role in lupus development (18). It is an anti-viral cytokine which is regulated by endosomal pattern recognition receptors (PRRs) such as TLRs or cytosolic PRRs like RIG-I like receptors (RLRs) (18, 19). It has the potential to break self-tolerance by inducing dendritic cell differentiation which in turn leads to activation of autoreactive T and B cells, thus linking innate and adaptive immune systems (20, 21). IFN-α is a heritable risk factor in SLE (22, 23) and some people who have received recombinant human IFN-α as a treatment for viral hepatitis C or malignancy have developed *de novo* SLE which resolves upon discontinuation of the IFN-α treatment (24). These data strongly support a causal role for IFN-α in SLE pathogenesis. Increased activity of IFN-α has been associated with presence of various SLE-associated autoantibodies, both anti-double-stranded DNA (anti-dsDNA) and anti-RNA-binding protein (anti-RBP) antibodies along with different organ involvement such as hematologic, renal, and central nervous systems (10, 25, 26). However, longitudinal studies have not confirmed the association between increases in IFIG expression and disease flare (27, 28). It seems that patients with high IFN-α have more severe disease and a higher rate of flare on average, but the changes in IFN-α levels in circulation do not correlate closely or quantitatively with changes in measures of disease activity over time.

Systemic lupus erythematosus is both more prevalent and more severe in African-American (AA) populations than in European-American (EA) populations, and disease manifestations are variable amongst different ancestral backgrounds (29–32). AA and Hispanic-American (HA) patients are likely to have more active SLE, with an earlier age at onset, than EA patients (31, 32). Anti-ribonucleoprotein (anti-RNP) and anti-Smith (anti-Sm) antibodies are more prevalent in AA patients than in EA and HA (30, 32), and a number of genetic variants are associated with autoantibody profiles in different ancestral groups (33, 34). Moreover, compared to EA patients, HA, and AA patients have a higher incidence of SLE-related renal disease, associated with anti-dsDNA and anti-RNP antibodies (31, 35). Additionally, some of the genetic factors associated with SLE are not shared between AA and EA patients (36–39). These data all support the idea that molecular and biological differences should exist in SLE patients of different ancestral backgrounds. We have shown that overall serum IFN-α activity is higher in SLE patients of non-European ancestry as compared to European ancestry, either directly or indirectly through an increased prevalence of anti-RBP antibodies (40, 41). In this study, we compare peripheral blood gene expression between AA and EA SLE patients taking into account the differences in autoantibody profile, and we find a striking difference in the activation of the IFN pathway between the two groups.

## Materials and Methods

### Patients, samples, and data collection

Serum samples were obtained from 149 female SLE patients from the University of Chicago Medical Center (UCMC) (*n* = 119) and NorthShore University Health System (*n* = 30). All cases fulfilled the American College of Rheumatology criteria for the diagnosis of SLE (1, 42), and the data regarding the presence or absence of these criteria as well as of SLE-associated autoantibodies [anti-nuclear antibodies (ANA), and anti-Ro, anti-La, anti-Sm, anti-RNP, and anti-dsDNA antibodies] were available for all patients. Forty-nine unrelated females who were screened by medical record review for the absence of autoimmune disease were used as controls. They were of similar age (*P* = 0.21) as the SLE cases. All subjects provided informed consent, and the study was approved by the institutional review boards at the Mayo Clinic and University of Chicago.

### Detection of autoantibodies

Antibodies to Ro, La, Sm, and RNP for all samples were measured by ELISA methods (INOVA Diagnostics, San Diego, CA, USA) at UCMC at the time of serum and RNA sampling, and standard clinical laboratory cutoff points were used to categorize them as positive or negative. Anti-dsDNA antibodies were measured using Crithidia luciliae immunofluorescence at UCMC, and detectable fluorescence was considered positive.

### Gene expression analysis

Thirty-three SLE cases and 16 age-matched controls were selected for microarray gene expression analysis. The cases were subdivided into AA and EA patients, and those with positive anti-RBP antibodies and those without as described in Table 1. Whole blood from the subjects was collected in PAX gene tubes (Qiagen), and RNA was purified in spin columns per manufacture recommendations. The RNA was analyzed on Affymetrix Gene 1.0 ST gene expression arrays, which were run in the University of Chicago Microarray Core facility. These intensity data were normalized through Affymetrix Expression Console software. Data from the microarray experiment have been deposited in the GEO database, accession number GSE50635.

**Table 1:** Samples and data collection for microarray analysis*.

		SLE cases**		Non-autoimmune controls
		RBP+	RBP−	
European-American	Female	8	8	8
African-American	Female	9	8	8

*There was no difference in age amongst the groups.

**All SLE cases fulfilled the ACR criteria for SLE. Anti-RBP (anti-Ro, anti-La, anti-Sm, and anti-RNP) antibodies were measured by ELISA, and anti-dsDNA antibody levels were measured using *Crithidia luciliae* immunofluorescence.

RBP+, anti-RNA-binding-protein (RBP) antibody positive; RBP−, RBP antibody negative.

Quantitative real-time PCR (qPCR) was used to validate the hypotheses generated from the microarray data with an independent replication cohort. The RNA of whole blood from 60 AA SLE patients, 47 anti-RBP antibody positive (RBP+), and 13 anti-RBP antibody negative (RBP−), and 56 EA SLE patients, 21 RBP+ and 24 RBP− along with 25 AA and 8 EA controls was purified using Qiagen RNeasy kit. cDNA was synthesized from total mRNA, and qPCR was used to measure relative transcript expression using SYBR Green dye on an ABI 7900HT thermal cycler.

### Statistical analysis

For each ancestry, the anti-RBP antibody status was used as a dichotomous variable, and each subgroup was compared to respective controls from the same ancestral background. Following normalization, the mean microarray gene expression values along with standard deviations were calculated for each subgroup and used to calculate the fold changes between subjects and controls. Values were compared between the groups using the two-tailed Student’s unpaired *t*-test. Similar comparisons were made with the qPCR data, but this time, all group results were expressed in medians and compared using Mann–Whitney tests as they did not follow Gaussian distributions. For both microarray and qPCR analyses, *P* values less than 0.05 were considered significant.

### Canonical pathway analysis

From the microarray data, the differentially expressed genes with a cutoff *P* value of 0.05 along with their respective fold changes were analyzed further through Ingenuity Pathway Analysis (IPA) (Ingenuity^®^ Systems, www.ingenuity.com) to compare the top canonical pathways amongst the sample groups (Table 1). The IPA canonical pathway analysis identified the pathways from the IPA Knowledge Base that were most significant to the data set. The significance of the association between the data set and the canonical pathway was measured in two ways: (1) A ratio of the number of molecules from the data set that map to the pathway divided by the total number of molecules that map to the canonical pathway was displayed. (2) Fisher’s exact test was used to calculate a *P* value determining the probability that the association between the genes in the dataset and the canonical pathway was explained by chance alone.

## Results

### Demographics and presence of ANA and anti-dsDNA antibodies

The average age of all subjects included in the microarray portion of the study was 43.5 ± 10.6 years. When the subjects and controls were divided into subgroups according to ancestry and the presence of anti-RBP antibodies (Table 1), there was no statistical difference in age amongst the subgroups including controls. There was lower prevalence in anti-dsDNA antibodies in RBP− AA patients (77% of RBP+ vs. 31% of RBP− had anti-dsDNA antibodies, *P* = 0.005). In EA subjects, this difference was much less pronounced, and was not statistically significant (66% of RBP+ vs. 54% of RBP− had anti-dsDNA antibodies, *P* = 0.4). This is in concordance with our previous large-scale analyses in SLE, in which we have also found that anti-RBP and anti-dsDNA antibodies are more correlated in AA as compared to EA SLE patients (40). All subjects were female and all tested positive for ANA.

### IFN-related canonical pathway activation in AA vs. EA SLE patients

Top 10 canonical pathways from each subgroup are shown in Table 2. Many immune system associated pathways were associated with the cases, and similar to previous studies, type I IFN-related pathways were the most differentially expressed when comparing cases vs. controls except in RBP− AA group. We examined in greater detail the six canonical pathways which were type I IFN-related across patients from the two different ancestral backgrounds studied. As shown in Table 3, the microarray analysis of all SLE cases vs. controls demonstrated all six IFN-related canonical pathways significantly involved. The same pattern was also observed for all EA cases vs. controls. However, to our surprise, the associations between the IFN-related canonical pathways and AA SLE cases were not as strong as only three out of six pathways were found to be significant. This is despite that fact that high circulating levels of type I IFN are more common in AA SLE patients (40).

**Table 2:** **Top 10 canonical pathways from each subgroup vs. matching controls through IPA from microarray data**.

All cases	All EA	All AA	EA RBP+	EA RBP−	AA RBP+	AA RBP−
EIF2 signaling	Interferon signaling	EIF2 signaling	Interferon signaling	Antigen presentation pathway	EIF2 signaling	Regulation of IL-2 expression in activated and anergic T lymphocytes
Interferon signaling	Antigen presentation pathway	Activation of IRF by cytosolic pattern recognition receptors	Activation of IRF by cytosolic pattern recognition receptors	OX40 signaling pathway	Regulation of eIF4 and p70S6K signaling	Glucocorticoid receptor signaling
Antigen presentation pathway	Role of pattern recognition receptors in recognition of bacteria and viruses	Angiopoietin signaling	Antigen presentation pathway	Autoimmune thyroid disease signaling	mTOR signaling	CD28 signaling in T helper cells
Activation of IRF by cytosolic pattern recognition receptors	Retinoic acid mediated apoptosis signaling	Regulation of eIF4 and p70S6K signaling	Role of pattern recognition receptors in recognition of bacteria and viruses	Allograft rejection signaling	Activation of IRF by cytosolic pattern recognition receptors	T cell receptor signaling
IL-12 signaling and production in macrophages	Activation of IRF by cytosolic pattern recognition receptors	Hypoxia signaling in the cardiovascular system	IL-15 production	Interferon signaling	Interferon signaling	Glycosphingolipid biosynthesis - globoseries
mTOR signaling	Graft-vs.-host disease signaling	Role of RIG1-like receptors in antiviral innate immunity	Retinoic acid mediated apoptosis signaling	Graft-vs.-host disease signaling	Apoptosis signaling	Biosynthesis of steroids
Role of pattern recognition receptors in recognition of bacteria and viruses	Dendritic cell maturation	mTOR signaling	Role of RIG1-like receptors in antiviral innate immunity	Cytotoxic T lymphocyte-mediated apoptosis of target cells	Colorectal cancer metastasis signaling	April mediated signaling
TNFR2 signaling	IL-15 production	Hereditary breast cancer signaling	Communication between innate and adaptive immune cells	Crosstalk between dendritic cells and natural killer cells	TNFR2 signaling	Reelin signaling in neurons
Production of nitric oxide and reactive oxygen species in macrophages	Autoimmune thyroid disease signaling	Role of PI3K/AKT signaling in the pathogenesis of influenza	Dendritic Cell maturation	Type I diabetes mellitus signaling	IL-8 signaling	Glycosphingolipid biosynthesis – neolactoseries
IL-15 production	Communication between innate and adaptive immune cells	TNFR2 signaling	Starch and sucrose metabolism	Dendritic cell maturation	P2Y purigenic receptor signaling pathway	Mitotic roles of polo-like kinase

IPA, ingenuity pathway analysis; EA, European-American; AA, African-American; RBP+, anti-RNA-binding-protein (RBP) antibody positive; RBP−, RBP antibody negative; eIF2, eukaryotic initiation factor 2; IRF, interferon-regulatory factor; mTOR, mammalian target of rapamycin; TNFR2, tumor necrosis factor receptor 2; eIF4, eukaryotic initiation factor 4; RIG1, retinoic acid-inducible gene 1; PI3K, phosphatidylinositol 3-kinase; OX40 = CD134.

**Table 3:** ***P* values for IPA IFN-related canonical pathways from microarray data**.

	All SLE cases (*n* = 33)	EA SLE cases (*n* = 16)	AA SLE cases (*n* = 17)
Interferon signaling	1.53 × 10^−^^10^	6.55 × 10^−^^12^	0.29
Activation of IRF by cytosolic pattern recognition receptors	6.14 × 10^−^^5^	2.74 × 10^−^^5^	8.46 × 10^−^^5^
Role of RIG1-like receptors in antiviral innate immunity	1.63 × 10^−^^4^	5.85 × 10^−^^7^	0.044
Role of PKR in interferon induction and antiviral response	0.020	0.038	0.091
Role of pattern recognition receptors in recognition of bacteria and viruses	0.0097	0.0064	0.0011
Communication between innate and adaptive immune cells	0.0098	0.0013	Not listed

IPA, ingenuity pathway analysis; IFN, interferon; SLE, systemic lupus erythematosus; EA, European-American; AA, African-American; IRF, interferon-regulatory factor; RIG1, retinoic acid-inducible gene 1; and PKR, protein kinase R.

### IFN-related canonical pathway activation is not seen in RBP− AA patients

To explore this further, we looked at the associations between IFN-related canonical pathways within SLE patient subgroups stratified by both ancestry and the presence or absence of anti-RBP antibodies. As shown in Table 4, all six type I IFN-related pathways were activated in both AA and EA RBP+ patients. The key pathway difference was found between AA and EA patients who were RBP−. RBP− EA patients demonstrated activation of all six IFN-related canonical pathways, whereas not a single type I IFN pathway was significantly involved in the RBP− AA patients.

**Table 4:** ***P* values from IPA IFN-related canonical pathways from microarray data**.

	EA RBP+	EA RBP−	AA RBP+	AA RBP−
Interferon signaling	5.8 × 10^−11^	10.6 × 10^−5^	1.3 × 10^−7^	0.063
Activation of IRF by cytosolic pattern recognition receptors	1.1 × 10^−6^	0.016	2.86 × 10^−8^	0.25
Role of RIG1-like receptors in antiviral innate immunity	2.0 × 10^−5^	0.030	0.0014	Not listed
Role of PKR in interferon induction and antiviral response	0.0073	0.042	9.0 × 10^−4^	Not listed
Role of pattern recognition receptors in recognition of bacteria and viruses	1.8 × 10^−4^	0.028	5.8 × 10^−4^	0.31
Communication between innate and adaptive immune cells	0.0043	0.0064	0.023	Not listed

IPA, ingenuity pathway analysis; IFN, interferon; EA, European–American; AA, African-American; RBP+, anti-RNA-binding-protein (RBP) antibody positive; RBP−, RBP antibody negative; IRF, interferon-regulatory factor; RIG1, retinoic acid-inducible gene 1; and PKR, protein kinase R.

### IFN-induced gene expression pathway diagrams in AA vs. EA patients

**Figure 1:**
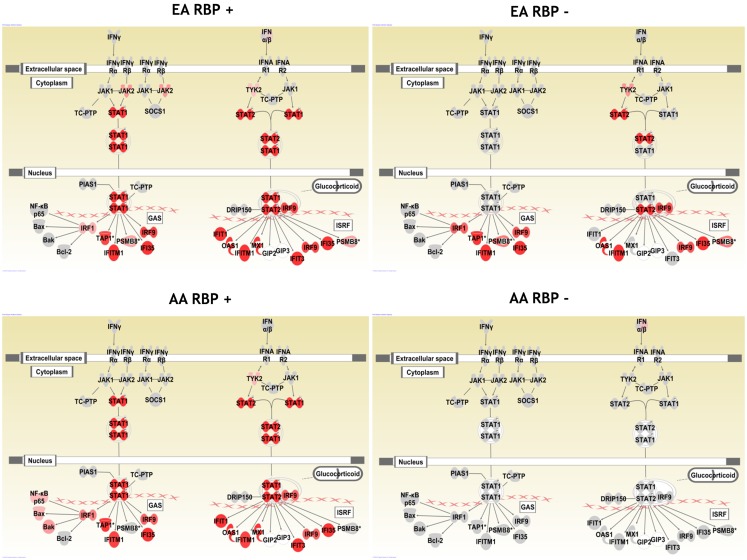
**Pathway diagram illustrating the type I and type II IFN pathways in SLE patient subsets**. Genes which are up-regulated are shaded red, with increasingly dark red shading indicating a greater degree of over-expression in cases as compared to controls of the same ancestral background. AA, African-American; EA, European-American; RBP+, anti-RNA-binding-protein (RBP) antibody positive; and RBP−, RBP antibody negative. Pictures generated using Ingenuity Pathway Analysis software.

In Figure 1, we show pathway diagrams generated in IPA software of the type I and type II IFN pathways, with genes that were up-regulated in cases vs. controls shaded red. It is striking that none of the genes illustrated downstream of the type I and type II IFN receptors are up-regulated in the RBP− AA patients, while in the RBP− EA patients, many IFN-induced genes are over-expressed. It is also interesting that STAT1 over-expression is observed in the RBP+ subjects regardless of ancestral background, and this is not observed in the RBP− patients from either ancestral background.

### Replication study confirms the dependence of IFN-induced gene expression upon presence of anti-RBP antibodies in AA patients, but not EA patients

**Figure 2:**
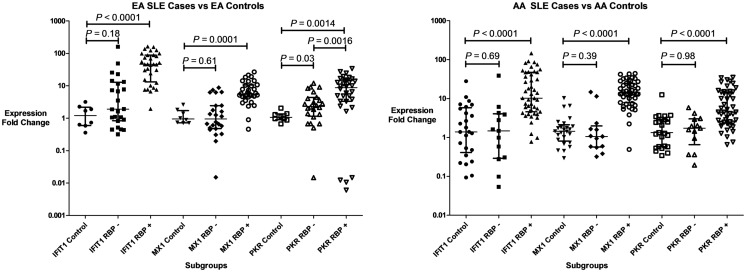
**Type I IFN-induced gene expression in SLE patient subgroups and controls**. Expression of three genes (IFIT1, MX1, and PKR) are shown in both patients with anti-RNA-binding protein antibodies (RBP+), and those who lack those antibodies (RBP−). Central tendency shown is a median, with error bars representing the interquartile range. *P* values generated by Mann–Whitney *U* test.

**Figure 3:**
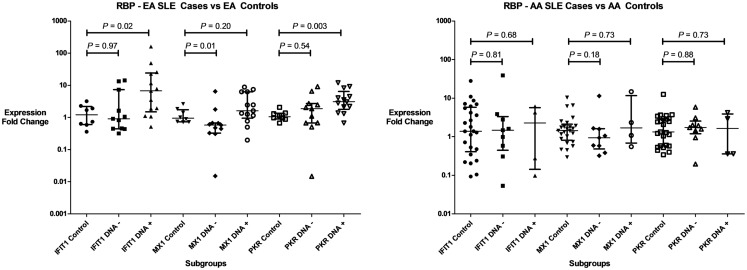
**Type I IFN-induced gene expression in RBP–SLE patient subgroups and controls in regards to of anti-dsDNA antibodies**. Expression of three genes (IFIT1, MX1, and PKR) are shown in both patients with anti-dsDNA antibodies (DNA+), and those who lack those antibodies (DNA−). Central tendency shown is a median, with error bars representing the interquartile range. *P* values generated by Mann–Whitney *U* test.

Three IFIGs (IFIT1, MX1, and PKR) were selected for qPCR analysis to replicate the microarray observation with regards to the association between anti-RBP antibodies and IFN-related gene expression across different ancestral backgrounds. These genes were quantified in whole blood mRNA from an independent cohort of 116 SLE patients and 33 controls. As shown in Figure 2, the pattern observed mirrors the microarray data. All three genes were up-regulated in both EA and AA RBP+ patients. In the RBP− patients, there is essentially no increase in IFN-induced gene expression in AA patients, while the expression of these genes, in particular PKR, is increased in the RBP− EA SLE patients. Because anti-dsDNA antibodies have been associated with high IFN-α (25, 26), it is important to determine whether anti-dsDNA antibodies are contributing to the induction of IFN-induced gene expression we observe in our RBP− EA patients. As noted above, the RBP− EA subjects were more likely to have anti-dsDNA antibodies than the RBP− AA subjects. When we looked at the RBP negative patients in the qPCR replication cohorts with regard to presence or absence of anti-dsDNA antibodies, there was no significant difference in IFIT1, MX1, or PKR over-expression in the AA subjects. In RBP− EA patients, however, over-expression of IFN-induced genes (IFIT1 and PKR) was observed in the anti-dsDNA antibody positive patients but not in the anti-dsDNA antibody negative group (Figure 3). Thus, in RBP− EA subjects, the anti-dsDNA antibody status did have an effect on expression of IFIGs, and thus anti-dsDNA antibodies are contributing to the IFN-induced gene expression in this group. Strikingly, anti-dsDNA antibodies had no impact upon IFN-induced gene expression in the RBP− AA group. This was somewhat unexpected and reinforces the idea of RBP antibody dependence in the AA ancestral background.

## Discussion

To our knowledge, this was the first study to show differential gene expression patterns in various subgroups of SLE patients stratified by ancestral background and presence or absence of anti-RBP antibodies. Through microarray whole genome expression with pathway analysis followed by independent qPCR validation, we demonstrated that activation of IFN-related pathways depended on presence of anti-RBP antibodies in AA patients, but not in EA patients. The results also support the model suggested by our previous study in which African ancestry increases the likelihood of SLE-associated autoantibody formation, leading to higher IFN-α activity (41). In the present study, we observe a similar dependence of IFN-induced gene expression upon anti-RBP antibodies in AA patients, and this is not shared with the EA patients, and this novel observation should be confirmed in larger cohorts.

Autoantibody immune complexes present in SLE patients have been implicated as major endogenous IFN-inducers, likely via the endosomal TLR and IFN regulatory factor pathways (43–45). Our data would suggest that the classical activation of IFN-related pathways observed in SLE patients is highly dependent upon anti-RBP antibodies in AA SLE patients, and this dependence is not shared by EA SLE patients. The additional IFN-pathway activation observed in EA subjects is partly due to the presence of anti-dsDNA antibodies. As shown in Figure 3 there are a number of anti-dsDNA and anti-RBP negative EA patients that show over-expression of IFN-induced genes, while in AA SLE patients lacking RBP antibodies, IFN-induced gene expression resembles the AA control population. This heterogeneity in the dependence of IFN-related pathways on autoantibody profile may reflect differential activation of the TLR pathway is SLE patients of different ancestral backgrounds. Anti-RBP antibodies would be expected to activate the RNA-sensing TLRs, while anti-dsDNA antibody immune complexes would be expected to activate TLR 9. Genetic variations in the TLR pathway genes such as IRF5 and IRF7 have been associated with risk of SLE, and with gain of function within the type I IFN pathway (46, 47). In our previous study looking at genetic variation at the IRF7/PHRF1 locus, we observed two different high IFN genetic effects in AA subjects, while we saw only one in EA subjects (46). This example demonstrates genetic diversity between world populations in the TLR/IRF system, and could support the idea that this pathway may more prominent in AA subjects and help to explain the findings we report here. Additionally, many of the genetic polymorphisms we have discovered that are associated with increased type I IFN in SLE patients differ between ancestral backgrounds (34, 48), and this would also support the idea that the pathway will be more or less prominent in different ancestral backgrounds.

There were EA SLE patients that had increased expression of IFN-induced genes who did not have either anti-RBP or anti-dsDNA autoantibodies. These data suggest that IFN-related pathways may be activated through different mechanisms in this ancestral background. All patients in our study had ANA, and it may be that other nuclear antigen/autoantibody complexes could have triggered the TLR/RLR system leading to IFN-pathway activation in these subjects. It is also possible that other molecules such as HMGB1, which can bind with immune complexes, may be activating an inflammatory cascade in plasmacytoid dendritic cells resulting in activation of IFN pathways (49). Another possibility is that there is an increased sensitivity to IFN signaling or a downstream activator of IFN-induced gene expression in these patients. We have observed some SLE patients in previous studies that have high IFN-induced gene activity in their PBMC with essentially normal circulating type I IFN activity from the same sample (50, 51).

This surprising diversity in IFN-pathway activation between different SLE patient subgroups is relevant to clinical care, as therapeutics directed at IFN or IFN-related pathways are being actively developed (52). It seems likely that these IFN-pathway targeting therapeutics will be characterized by heterogeneity in treatment response, and our results may suggest some groups that are likely to be better responders that could be predicted without having to run a gene expression chip prior to therapy. The RBP− AA group is very interesting in this regard, as it seems that this patient group may represent a distinct subset of SLE patients which is not as IFN-dependent as other groups of SLE patients. This may represent a significant difference in disease pathogenesis, which could be important in planning targeted therapies.

## Conclusion

Systemic lupus erythematosus is a heterogeneous disease with differences in disease incidence, clinical manifestations, serological findings, and genetic risk factors between ancestral backgrounds (7, 29–32, 36–39). Perhaps it is not very surprising to find heterogeneity in the activation of molecular pathways between ancestral groups, and it seems likely that different pathogenic factors will be relevant in RBP− AA patients as compared to the RBP+ SLE patients. SLE is a complex autoimmune disease, and understanding heterogeneity in the molecular pathogenesis in lupus will be crucial in informing therapeutic and diagnostic strategies. This study demonstrates the relevance of careful patient characterization and including patients from more than one ancestral background in biological studies of SLE.

## Author’s Contributions

Kichul Ko participated in the design of the study and its coordination, screened for cases and controls, lead the statistical analysis, and drafted the manuscript. Yelena Koldobskaya helped screen for cases and controls, and collected microarray data. Elizabeth Rosenzweig helped generate qPCR data. Timothy B. Niewold was the senior scientist overseeing the study and helped with study design, statistical analysis, and manuscript drafting.

## Conflict of Interest Statement

The authors declare that the research was conducted in the absence of any commercial or financial relationships that could be construed as a potential conflict of interest.

## Abbreviations

AA, African-American; ANA, antinuclear antibodies; Anti-dsDNA, anti-double-stranded DNA; Anti-RBP, anti-RNA-binding protein; Anti-RNP, anti-ribonucleoprotein; Anti-Sm, anti-smith; EA, European-American; HA, Hispanic-American; IFIGs, IFN-inducible genes; IFN, interferon; IPA, ingenuity pathway analysis; PRRs, pattern recognition receptors; qPCR, quantitative real-time PCR; RBP+, anti-RBP antibody positive; RBP−, anti-RBP antibody negative; RLRs, RIG-I like receptors; SLE, systemic lupus erythematosus; TLR, toll-like receptor; UCMC, University of Chicago Medical Center.
